# Computational Intelligence Modeling of the Macromolecules Release from PLGA Microspheres—Focus on Feature Selection

**DOI:** 10.1371/journal.pone.0157610

**Published:** 2016-06-17

**Authors:** Hossam M. Zawbaa, Jakub Szlȩk, Crina Grosan, Renata Jachowicz, Aleksander Mendyk

**Affiliations:** 1 Faculty of Mathematics and Computer Science, Babes-Bolyai University, Cluj-Napoca, Romania; 2 Faculty of Computers and Information, Beni-Suef University, Beni-Suef, Egypt; 3 Department of Pharmaceutical Technology and Biopharmaceutics, Jagiellonian University Medical College, Krakow, Poland; 4 College of Engineering, Design and Physical Sciences, Brunel University, London, United Kingdom; Laurentian, CANADA

## Abstract

Poly-lactide-co-glycolide (PLGA) is a copolymer of lactic and glycolic acid. Drug release from PLGA microspheres depends not only on polymer properties but also on drug type, particle size, morphology of microspheres, release conditions, etc. Selecting a subset of relevant properties for PLGA is a challenging machine learning task as there are over three hundred features to consider. In this work, we formulate the selection of critical attributes for PLGA as a multiobjective optimization problem with the aim of minimizing the error of predicting the dissolution profile while reducing the number of attributes selected. Four bio-inspired optimization algorithms: antlion optimization, binary version of antlion optimization, grey wolf optimization, and social spider optimization are used to select the optimal feature set for predicting the dissolution profile of PLGA. Besides these, LASSO algorithm is also used for comparisons. Selection of crucial variables is performed under the assumption that both predictability and model simplicity are of equal importance to the final result. During the feature selection process, a set of input variables is employed to find minimum generalization error across different predictive models and their settings/architectures. The methodology is evaluated using predictive modeling for which various tools are chosen, such as Cubist, random forests, artificial neural networks (monotonic MLP, deep learning MLP), multivariate adaptive regression splines, classification and regression tree, and hybrid systems of fuzzy logic and evolutionary computations (fugeR). The experimental results are compared with the results reported by Szlȩk. We obtain a normalized root mean square error (NRMSE) of 15.97% versus 15.4%, and the number of selected input features is smaller, nine versus eleven.

## 1 Introduction

Poly-lactide-co-glycolide (PLGA) is a copolymer of lactic and glycolic acid. Hydrolysis of PLGA ester bonds in the human body leads to monomers, which can be introduced in Krebs cycle because they occur naturally. Therefore, PLGA is considered as a biodegradable and biocompatible polymer with minimal toxicity. A wide range of PLGA products is used as matrices for drug delivery systems such as microspheres, implants, in-situ gelling solutions, and medical devices (sutures, stents) [1]. Together with its biocompatibility, PLGA drug-protective properties as carriers are exploited i.e. for peptide drugs, DNA, RNA [2].

Despite an enormous amount of research focusing on PLGA microspheres, the complexity of such multi-compartment systems requires a more thorough understanding of their behavior. All types of variables can affect dissolution. It depends not only on polymer properties but also on drug type, particle size, the morphology of microspheres, release conditions, etc. [3]. Extracting knowledge from already gathered data is one approach to filling the research gap. Therefore, in this work, we have used existing data sets to discover complex relationships of releasing drug from PLGA matrices.

This study was conducted with the scope of analyzing the potential of some bio-inspired computational techniques for modeling experimental data coming from pharmaceutical sciences, where there is a need to find crucial variables governing the behavior of pharmaceutical dosage forms, in particular, PLGA microspheres. During the last decade, research for elucidation of macromolecular drugs release mechanism from PLGA-based drug delivery systems were in focus [4–7]. Most of the work is based on mathematical modeling [4, 5, 8, 9]. Only a few are directly related to the usage of heuristic computational techniques for knowledge extraction [10, 11].

Due to the recently introduced Process Analytical Technology (PAT) initiative [12] and the concept of Quality by Design (QbD) [13], there is a need to introduce to pharmaceutical industry multivariate, robust modeling techniques suitable for identification of crucial variables governing the analyzed processes. Moreover, PAT urges the need for thorough understanding of relationships responsible for pharmaceutical formulation behavior.

In QbD, this conforms with the selection of critical quality attributes (CQAs) and critical process parameters (CPPs), where an influence of formulation composition and technological process variables on desired quality of developed formulation should be taken into consideration. Numerous factors, like raw materials stability, lactide to glycolide acid ratio in polymers and technological process play a significant role in formulation quality. Thanks to the development of modern analytical techniques the number of potential CQAs and CPPs is counted in hundreds or even thousands of factors. In order to minimize the risk of inadequate quality of formulation, appropriate technology, and raw material is selected and controlled by careful choice of the minimal set of CQAs and CPPs. Reduction of control variables is performed both for the sake of clarity of reasoning and numerical stability of the developed models. For the latter, a so-called “curse of dimensionality” [14] is a rationale for simplification of the models. It is especially applicable in the holistic approach [15] integrating CQAs and CPPs into the single model. Such solution is known for improving knowledge management and is a natural domain for application of computational intelligence (CI) tools [15]. Data-driven models based on CI are developed without a priori assumption and based on automatically acquired knowledge could be used for selection of CQAs and CPPs in an empirical manner. However, for this task, a robust and efficient feature selection method is needed, which is in the focus of this work concentrated on the in vitro dissolution profiles of macromolecules from PLGA microspheres. Our rationale is that CI tools could select some of CQAs and CPPs relevant to this endpoint.

## 2 Materials and Methods

### 2.1 Materials

**Fig 1:**
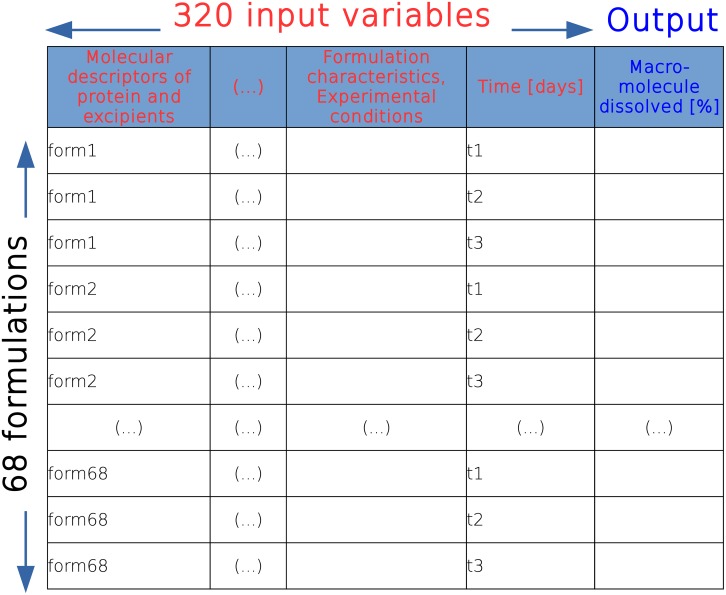
Construction of the data set.

For consistency in comparison of the results for both approaches, original data was extracted from publication and the structure of the data was retained as in Szlȩk et al. [10]. In brief, the data was gathered after reviewing about 200 scientific publications. The extracted data consists of drug release profiles of 68 PLGA formulations from 24 publications. Originally, the input vector consisted of 320 variables (molecular descriptors of protein, excipients, formulation characteristics, and the experimental conditions) and 745 time points (records). All data were of numeric format with continuous values, except variables such as “Production method” and “Lactide to glycolide ratio” which took discrete values (1, 2, 3, etc.). The amount of the drug substance released (Q) was the only dependent variable as shown in Fig 1, its values were ranging from 0 to 100%. Data set which were used during the modeling have been uploaded on SourceForge [16].

Data set was split according Data set was split according to the 10-fold cross-validation scheme, each time excluding all data belonging to the particular formulation, thus simulating the real application of the model forced to predict the dissolution behavior of the unknown formulation [17]. Preliminary studies indicated that linear scaling of the data from 0.1 to 0.9 did not lower the prediction error, thus the data without normalization was used. We did not use external validation data set due to limited data available in the literature. In order to predict a dissolution for all the proposed models, the data from publication must have: protein incorporated into PLGA microsphere, its dissolution profile, protein with known structure to obtain chemical descriptors, process parameters etc. Therefore publications fulfilling all above criteria are scarce.

### 2.2 Procedure for crucial variables selection

The identification of smallest possible feature subset is a complex search problem, proved to be NP-Complete [18]. Therefore, an exhaustive examination of all possible subset combinations of features is not tractable for large feature sets. For our case, we have 2^320^ possible subsets, which is roughly 2.13*e*^96^ sets of features to be investigated. A reasonable approach is to use approximation algorithms or heuristics. We will use randomized search, specifically bio-inspired randomized search algorithms because there is evidence in the literature showing that they are effective for features selection [19–22]. These algorithms evolve a population of subsets of features towards those that meet specified criteria. We will try here four different bio-inspired algorithms [23]. In addition to these methods, we use the well known LASSO algorithm for comparison purposes.

Once a subset of features has been selected, the performance of a predictive model on the selected attributes is tested. We selected a number of well-known predictive tools such as Cubist, random forests, artificial neural networks (monotonic MLP, deep learning MLP), multivariate adaptive regression splines (MARS), classification and regression tree (CART), and hybrid systems of fuzzy logic and evolutionary computations (fugeR). In fact, the predictive models are not just used at the end of the selection process, but their are build into the evaluation (fitness) function of each of the feature selection methods. The feature selection methods improve a randomly generated solution iteratively, and each time an improvement is suggested, its quality is assessed by a predictive model.

#### 2.2.1 Feature selection models

The proposed feature selection models are needed to find a *minimal* subset of input variables (features) from several hundred that *minimize* the model prediction error. The search space represents each variable as an individual dimension, and the span of each dimension ranges from 0 to 1 and hence requires an inventive searching method to find optimal subset in the huge search space that minimizes the fitness function. We define the fitness/objective function as follows:

$$\begin{array}{c} ↓Fitness=\alpha \;P+\;\beta \;\frac{|R|}{|C|}, \end{array}$$ (1)

where *P* is the error of the prediction model, *R* is the size of selected variable subset, *C* is the total number of variables in the data set, *α* and *β* are two parameters corresponding to the importance of prediction performance and subset size, *α* ∈ [0, 1] and *β* = 1− *α*.

Below we briefly describe the algorithms used for selecting the features. The main parameters of the bio-inspired techniques are given in Table 1, but there are other parameters specific to each method which is set either according to domain-specific knowledge or based on trial and error on small simulations.

**Table 1:** Parameter setting for the bio-inspired methods.

Parameter	Value(s)
K for cross validation	10
No. of search agents	8
No. of iterations	100
Problem dimension	Number of features in the data
Search domain in binary algorithms	{0, 1}
Search domain in continuous algorithms	[0, 1]
*α* parameter in the fitness function	0.99
*β* parameter in the fitness function	0.01

1. *Antlion optimization*.
  Antlion optimization (ALO) is a bio-inspired optimization algorithm proposed by Mirjalili [24]. ALO algorithm imitates the chasing mechanism of antlions in nature. The pseudocode of antlion optimization (ALO) is given in Algorithm 1. Complete clarification of the main steps and details about the role of ALO’ parameters can be found in [24].
2. *Binary antlion optimization*.
  The optimization algorithms in continuous mode usually find a feature subset that maximizes the classifier performance where search agents are positioned in a *d*-dimensional search space at positions over the interval [0, 1]. In binary mode, the search space is much more limited as only two values {0, 1} are allowed for each dimension and hence is expected that the optimizer will perform better. Binary antlion optimization (BALO) is an extension of ALO to work in binary mode. While in ALO antlions and ants are continuously changing their positions to any point in the space, in BALO, the solutions are restricted to the binary {0, 1} values. This modification of ALO adapts and performs very well for the feature selection task. The pseudocode of a binary version of antlion optimization (BALO) is presented in Algorithm 2. More details can be found in [25].
  **Algorithm 1**: Antlion optimization (ALO) algorithm.
  1: **Input**: *N* number of ant lions, *n* number of ants, *N*_*Iter*_ maximum number of iterations.
  2: **Output**: The optimal ant lion binary position and its fitness value.
  3: Randomly initialize a population of ant positions and a population of antlion positions.
  4: Calculate the fitness of all the ants and antlions.
  5: Find the fittest antlion (elite).
  6: *t* = 0.
  7: **while** *t* ≤ *T* **do**
  8:  **for all** *Ant*_*i*_ **do**
  9:   Select an antlion using Roulette wheel.
  10:   Slide ants toward the antlion.
  11:   Create a random walk for the *Ant*_*i*_ and normalize it
  12:  **end for**
  13:   Calculate the fitness of all ants.
  14:   Replace an antlion with its corresponding ant; if the ant becomes fitter).
  15:   Update the elite; if an antlion becomes fitter than the current elite.
  16:   *t* = *t*+1
  17: **end while**
  18: Select the elite antlion *elite* and its fitness.
  **Algorithm 2**: Binary antlion optimization (BALO) algorithm
  1: **Input**: *N* number of antlions, *n* number of ants, *N*_*Iter*_ maximum number of iterations.
  2: **Output**: Optimal antlion binary position (*Elite*) and its fitness value.
  3: Initialize a population of *n* ants′ positions at random ∈0,1.
  4: Initialize a population of *N* antlions′ positions at random ∈0,1.
  5: Calculate the fitness of all ants and antlions.
  6: Find the fittest ant lion (elite).
  7: **While** Stopping criteria do not meet **do**
  8:  Calculate the mutation rate (*r*) given the random walk step size and current iteration number.
  9:  Calculate the fitness of all ants.
  10:  Replace an antlion with its corresponding ant; if it becomes fitter (Catching Prey).
  11:  Update elite; if an antlion becomes fitter than it.
  12: **end while**
  13: Select the elite antlion (elite) and its fitness.
3. *Grey wolf optimization*.
  Grey Wolf Optimization (GWO) is bio-inspired heuristic optimization algorithm that imitates the way in which wolves search for food and survive by avoiding their enemies [26]. Grey wolves are social animals that live in groups, and the pack size contains between 5 to 12 wolves on average. In the computational model, *α* is the fittest solution, *β* and *δ* are the second and third best solutions. The hunting process is guided by *α*, *β*, and *δ* while the *ω* follow them. The algorithm is outlined in Algorithm 3, with more details being available in [27, 28].
4. *Social spider optimization*.
  Social Spider Optimization (SSO) algorithm mimics the social behavior of the spider colony in nature [29]. SSO is swarm-based and consists of two main components: social members and communal web [30]. Social spider optimizer is formally presented in algorithm 4, with more details about the algorithm implementation available in [29, 31, 32].
  **Algorithm 3**: Grey wolf optimization (GWO) algorithm
  1: **Input**: *N* number of grey wolves, *N*_*Iter*_ maximum number of iterations.
  2: **Output**: Optimal grey wolf position and its fitness value.
  3: Initialize a population of *N* positions of the grey randomly.
  4: Find the *α*, *β*, and *δ* solutions based on their fitness values.
  5: **while** Stopping criteria does not meet **do**
  6:  **for all** *Wolf*_*i*_ ∈ *pack* **do**
  7:   Update current grey wolf′s position.
  8:  **end for**
  9:  Update coefficient vectors.
  10:  Evaluate the positions of individual wolves.
  11:  Update *α*, *β*, and *δ*.
  12: **end while**
  13: Select the optimal grey wolf position and its fitness.
  **Algorithm 4**: Social spider optimization (SSO) algorithm
  1: **Input**: *N*_*f*_ number of female spiders, *N*_*m*_ male spiders, *PF* attraction threshold, *N*_*Iter*_ maximum number of iterations.
  2: **Output**: Optimal social spider position and its fitness value.
  3: **while** *t* ≤ *N*_*Iter*_ **do**
  4:  Randomly initialize the female spiders.
  5:  Randomly initialize the male spiders.
  6:  Evaluate the fitness (weight) of each spider.
  7:  Calculate the female cooperative operator.
  8:  **for** *i* = 1;*i* < = *N*_*f*_;*i*++ **do**
  9:   Calculate the *Vibc*_*i*_ and *Vibb*_*i*_.
  10:  **end for**
  11:  Calculate the male cooperative operator.
  12:  Calculate the *Vibf*_*i*_.
  13:  Perform the mating operation.
  14:  Update the best solution; if a spider becomes fitter than the best.
  15: **end while**
  16: Produce the best spider position and its fitness.

Besides the bio-inspired methods above, we also used Least absolute shrinkage and selection operator (LASSO) for comparison purposes. Tibshirani has introduced an attractive LASSO method for shrinkage and variable selection in 1996 [33]. LASSO method applies the penalty concept; the optimization should depend on the quadratic program (QP) or non-linear general program that is recognized to be computationally expensive. LASSO performs better prediction accuracy by shrinkage as the ridge regression. Therefore, LASSO is a useful tool to accomplish the reduction (shrinkage) and variable selection operations simultaneously [34].

### 2.3 Predictive models

1. *Cubist*: is a package which implements Cubist rule-based predictive decision trees development firstly proposed by Quinlan [35]. Cubist models introduce linear equations at their terminal branches; therefore, they can predict numeric values. The maximum number of rules was fixed at 100, the number of committees was set from one to 100. The extrapolation parameter, which controls the estimation ability of created models beyond the original observation range, was set to 100. The sample parameter, which is a percentage of the randomly selected data set for model building, was established at zero, which means that no data subsampling was employed, and all the models were built on previously prepared 10cv data sets [36].
2. *Random Forest (RF)*: creates an ensemble of decision trees using random inputs. Package randomForest of R environment was used [37]. It implements the Fortran code proposed by Breiman and Cutler [38]; therefore, it is suitable both for classification and regression problems. Similar to classification, regression forest is formed by growing trees depending on a random vector, but instead of categorical response in case of classification, the tree predictor takes on numerical values. Then the output predictor is formed by taking the average over all trees [39]. During model development, the following parameters have been used: number of randomly selected variables at each split was between 1 and half the size of a vector (mtry), maximum number of nodes was set between 10 and 500 (maxnodes), and number of trees was set from 10 to 500 (ntree).
3. *Monmlp (monotonic multilayer perceptron)*: [40, 41] is used to take advantage of the learning without back-propagation. Thus, monotonicity has been turned off. All of the prepared models had two hidden layers, each one numbering from 2 to 20 nodes. The hidden layer uses hyperbolic tangent (tansig) transfer function, and the output layer uses linear function applied. Ensemble systems were employed and consisted of ten neural networks. Variables have been scaled linearly from 0.1 to 0.9. Epoch has been set from 50 to 1,000. The “trials” parameter has been set to 5 to avoid getting stuck in local minima.
4. *Deep learning neural networks (h2o)*: we observed that, in order to properly train neural networks of complicated functions, noisy, or nonlinear data, deep architectures may be needed. The term “deep architecture” refers to a neural network composed of multiple hidden layers with many neurons within each layer. Deep learning neural networks are used to solve complex problems by introducing combinations of simpler solutions. Therefore, those systems can operate in real-world environments [42]. Hyperbolic tangent has been used as activation function of choice. Epochs varied from 1,000 to 10,000,000. Neural nets consisted of two to eight hidden layers with two to two hundred nodes per layer. Overall more than 250 architectures have been trained and tested [43].
5. *fugeR*: an idea of applying genetic algorithms in the field of fuzzy modeling appeared in the early nineties of last century [44]. Fuzzy systems gain the advantages of evolutionary techniques to develop a model based on large, and often complex, data sets. At the same time retaining its simplicity of representing data as linguistic variables and describing relations between variables by conditional rules. The package fugeR is designed for training fuzzy systems based on evolutionary algorithms. A maximum number of rules developed during training varies from 10 to 500. Maximum variables per rule have been set from 4 to 9. A number of generations considered are 50, 100, 200, 500 or 1000, and the population was either 100 or 500. The elitist parameter was set to be 20% out of every generation [45].
6. *Classification and regression tree (CART)*: was used in order to compare with more sophisticated models such as the random forest. CART was first introduced by Breiman et al. [46]. CART is a machine-learning method for constructing prediction models from the data. The models are trained by partitioning the multidimensional space and fitting a simple prediction model within each partition. When a modeling is done, the results can be represented graphically as a decision trees. Regression trees, which were used in this article, are designed for dependent variables that take continuous or discrete values. In this case, the prediction error is typically measured by the squared difference between the observed and predicted values [47].
7. *Multivariate adaptive regression splines (MARS)*: was introduced by Jerome H Friedman in 1991 [48]. MARS model is a weighted sum of constant and basic functions multiplied by the coefficients. Basic function in MARS models is so called hinge function. The model development is usually composed of 2 steps. In the first step, the model is created from the single intercept and is extended iteratively by adding pairs of hinge functions. This process leads to a reduction of training error and produces the overfitted model. Therefore, during the next step redundant, basic functions are removed from the model to improve generalization ability of the final model [49]. In this work, earth package for R environment was implemented in presented work as an example of multivariate adaptive regression splines method [50].

## 3 The proposed system evaluation

The measures described in the following section are used as indicators of the quality of solutions for the computational models. Root mean square error (RMSE) is used by both the feature selection and the predictive models. Normalized RMSE is used for clarity of presentation and comparability with other works. Each algorithm has been applied 20 times with random positioning of the search agents. Repeated runs of the optimization algorithms were used to test their convergence capability.

### 3.1 Performance metrics

We use the following notations:

- *obs*_*i*_ and *pred*_*i*_ are the observed and predicted values respectively;
- *X*_*max*_ and *X*_*min*_ are the maximum and minimum observed values respectively;
- *μ* is the mean of the observed values;
- *n* is total number of samples;
- *i* is the index of a sample in the data set.

The indicators (measures) used to compare the different algorithms are as follows:

1. *Root mean square error (RMSE)*: measures the average squared root errors, the error being the difference between the observed output and the predicted output, as given in Eq (2):
  $$\begin{array}{c} RMSE=\sqrt{\frac{\sum_{i=1}^{n}{\left(obs_{i}-pred_{i}\right)}^{2}}{n}} \end{array}$$ (2)
2. *Normalized root mean square error (NRMSE)*: is the normalized root mean square error (NRMSE): as in Eq (3).
  $$\begin{array}{c} NRMSE=\frac{RMSE}{X_{max}-X_{min}}\cdot 100\% \end{array}$$ (3)
3. *R-squared (R^2^)*: is the proportion of variability in a data set that is accounted for by a statistical model as given in Eq (4).
  $$\begin{array}{c} R^{2}=1-\frac{\sum_{i=1}^{n}{\left(obs_{i}-pred_{i}\right)}^{2}}{\sum_{i=1}^{n}{\left(obs_{i}-\mu \right)}^{2}} \end{array}$$ (4)

#### 3.1.1 Results and discussions

**Fig 2:**
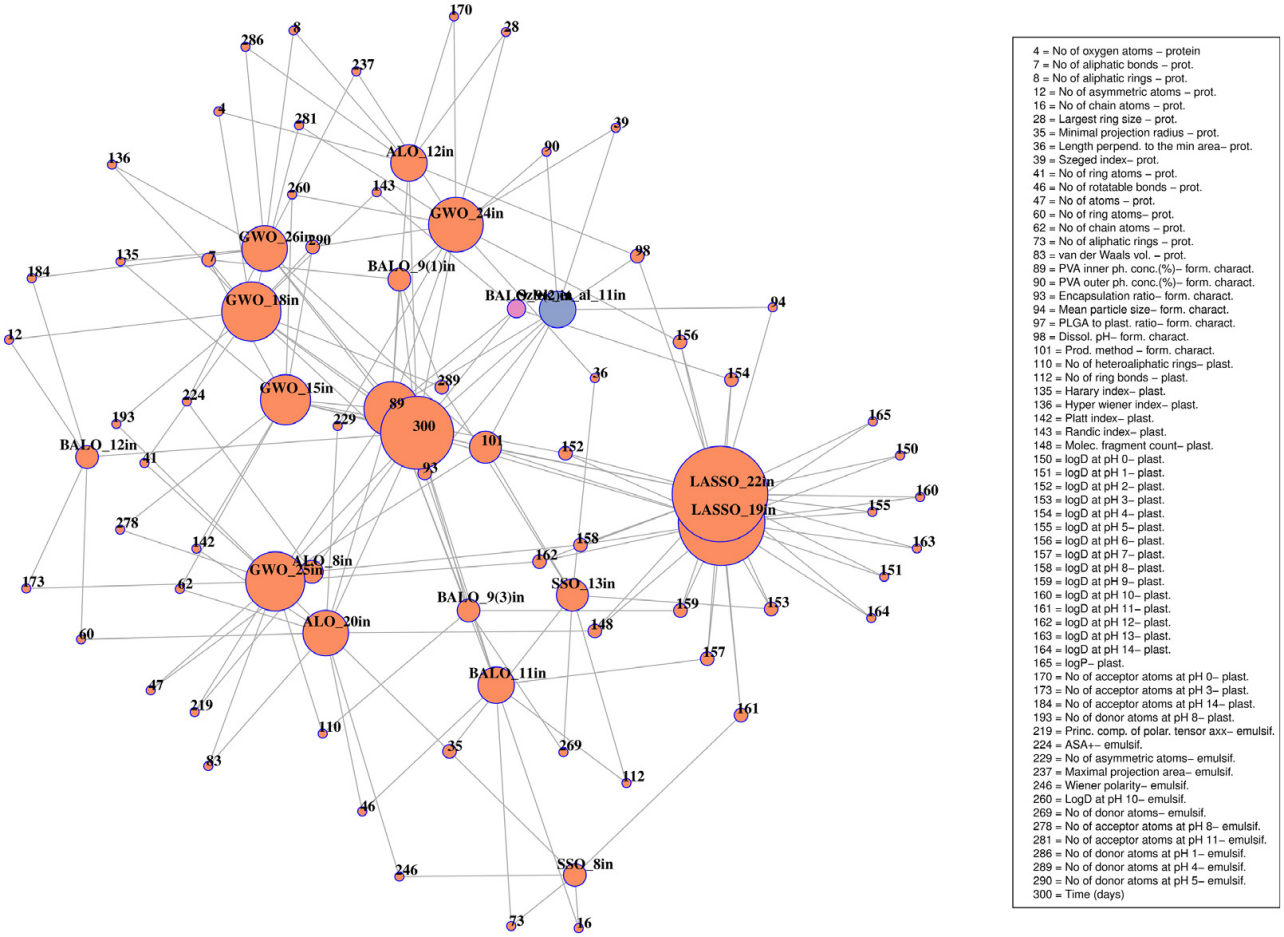
Graph showing frequencies of occurrence together with connections between selected vectors of features. Connections are drawn when variable is present in at least two subsets of features.

Overall, regarding all methods and 10-cv (cross-validation) sets, nearly 18,000 models were trained and tested. Feature selection models selected various combinations of features as presented in Table 2. Fig 2 shows the frequencies of features regarding all subsets of features selected by ALO, BALO, GWO, and SSO (orange circles) together with features obtained by Szlȩk et al. [10] (blue circle). Connections are drawn when a variable is present at least in two subsets of features. NRMSE varied from 31.1% to 15.9%. Random Forest algorithm yielded the lowest error; therefore, it was used for selecting optimal inputs vector as in Table 3.

**Table 2:** PLGA features selected by the optimization algorithms.

FS method	No. Inputs	Input Labels
**ALO**	8	**Protein descriptor**: Randic index, No of atoms; **Plasticizer descriptor**: logD at pH 8, No of acceptor atoms at pH 9; **Emulsifier descriptor**: No of rings, Principal component of polarizability tensor axx, Water accessible surface area of all atoms with positive partial charge; **Assay conditions**: Time (days)
**ALO**	12	**Protein descriptor**: No of asymmetric atoms, Largest ring system size, No of ring atoms, No of ring bonds, No of aliphatic atoms, No of bonds; **Plasticizer descriptor**: No of stereoisomers, No of acceptor atoms at pH 3, No of acceptor atoms at pH 14; **Emulsifier descriptor**: No of rotatable bonds, No of donor atoms at pH 7; **Assay conditions**: Time (days)
**ALO**	20	**Protein descriptor**: Dreiding energy, Maximal projection area, No of rotatable bonds, No of ring atoms, No of chain atoms, No of aliphatic bonds, van der Waals volume, Formulation characteristics: PVA inner phase concentration (%), Inner phase volume (mL); **Plasticizer descriptor**: No of aliphatic atoms, Platt index, count, No of hydrogen bond donors, No of acceptor atoms at pH 10, No of donor atoms at pH 10; **Emulsifier descriptor**: No of bonds, Water accessible surface area of all polar atoms, No of asymmetric atoms, Wiener polarity; **Assay conditions**: Time (days)
**BALO**	9(1)	**Protein descriptor**: No of hydrogens, No of heteroaromatic rings, No of rings, No of heteroaliphatic rings; Formulation characteristics: PVA inner phase concentration (**Plasticizer descriptor**: No of heteroaliphatic rings, logD at pH 9; **Emulsifier descriptor**: No of hydrogen bond donors; **Assay conditions**: Time (days)
**BALO**	9(2)	**Protein descriptor**: -; Formulation characteristics: PVA inner phase concentration (%); **Plasticizer descriptor**: Minimal projection radius, Randic index, logD at pH 4; **Emulsifier descriptor**: No of aromatic atoms, Szeged index, logD at pH 9, No of donor atoms at pH 8; **Assay conditions**: Time (days)
**BALO**	9(3)	**Protein descriptor**: No of aliphatic bonds, No of rings, No of aromatic atoms; Formulation characteristics: PVA inner phase concentration (%), PVA outer phase concentration (%); **Plasticizer descriptor**: -; **Emulsifier descriptor**: No of ring bonds, logD at pH 3, No of donor atoms at pH 4; **Assay conditions**: Time (days)
**BALO**	11	**Protein descriptor**: No of chain atoms, No of rotatable bonds, No of aliphatic rings; Formulation characteristics: PVA inner phase concentration (%), Encapsulation ratio; **Plasticizer descriptor**: No of ring bonds, logD at pH 7; **Emulsifier descriptor**: Dreiding energy, No of acceptor atoms at pH 10, No of acceptor atoms at pH 12; **Assay conditions**: Time (days)
**BALO**	12	**Protein descriptor**: No of oxygen atoms, No of aliphatic rings, Largest ring size, No of chain bonds; Formulation characteristics: PVA inner phase concentration (%), PLGA concentration (%), Dissolution pH; **Plasticizer descriptor**: No of acceptor atoms at pH 0, No of donor atoms at pH 12; **Emulsifier descriptor**: No of donor atoms at pH 1, No of donor atoms at pH 14; **Assay conditions**: Time (days)
**GWO**	15	**Protein descriptor**: No of aliphatic bonds, No of rings of atom, No of bonds, No of chain atoms; Formulation characteristics: PVA inner phase concentration (%), Production method; **Plasticizer descriptor**: Harary index, Platt index, logD at pH 2, No of donor atoms at pH 4; **Emulsifier descriptor**: logD at pH 10, No of acceptor atoms at pH 1, No of acceptor atoms at pH 8, No of donor atoms at pH 5; **Assay conditions**: Time (days)
**GWO**	18	**Protein descriptor**: No of oxygen atoms, No of aliphatic bonds, No of asymmetric atoms, Platt index, No of ring atoms, Ring system count, No of aromatic rings; Formulation characteristics: PVA inner phase concentration (%), Encapsulation ratio; **Plasticizer descriptor**: molecular, Hyper wiener index, Randic index, No of donor atoms at pH 8; **Emulsifier descriptor**: No of molecule fragments, No of donor atoms at pH 11, No of donor atoms at pH 4, No of donor atoms at pH 5; **Assay conditions**: Time (days)
**GWO**	24	**Protein descriptor**: Largest ring size, Minimal projection area, Length perpendicular to the min area, Szeged index, No of heteroaliphatic rings, No of chain atoms; Formulation characteristics: PVA inner phase concentration (%); **Plasticizer descriptor**: Polar surface area, No of molecule fragments, logD at pH 6, bpKa1, No of acceptor atoms at pH 0; **Emulsifier descriptor**: No of aliphatic atoms, principal component of polarizability tensor ayy, principal component of polarizability tensor ayy, No of asymmetric atoms, No of chiral centers, Maximal projection area, logD at pH 6, logD at pH 10, No of acceptor atoms at pH 7, No of acceptor atoms at pH 11, No of donor atoms at pH 5; **Assay conditions**: Time (days)
**GWO**	25	**Protein descriptor**: Cyclomatic number, Minimal projection radius, No of ring atoms, Atom count, No of aromatic atoms, Largest ring size, van der Waals volume; Formulation characteristics: PVA inner phase concentration (%), Dissolution additive concentration (%), Production method; **Plasticizer descriptor**: No of heteroaliphatic rings, Water accessible surface area of all hydrophobic atoms, Dreiding energy, logD at pH 12, No of acceptor atoms at pH 3, No of donor atoms at pH 8, No of donor atoms at pH 9, No of donor atoms at pH 14; **Emulsifier descriptor**: principal component of polarizability tensor axx, ASA, Water accessible surface area of all hydrophobic atoms, No of acceptor atoms at pH 8, No of acceptor atoms at pH 9, No of donor atoms at pH 10; **Assay conditions**: Time (days)
**GWO**	26	**Protein descriptor**: No of aliphatic rings, No of chiral centers, Balaban index, Hetero No of rings, HeteroNo of aromatic rings; Formulation characteristics: PVA inner phase concentration (%), PVP molecular weight, Production method; **Plasticizer descriptor**: Largest ring size, Harary index, Hyper wiener index, Szeged index, bpKa2, No of acceptor atoms at pH 4, No of acceptor atoms at pH 14; **Emulsifier descriptor**: No of aliphatic bonds, No of aliphatic rings, Water accessible surface area of all atoms with positive partial charge, Water accessible surface area of all atoms with negative partial charge, Maximal projection area, logD at pH 8, logD at pH 14, logP, bpKa2, No of donor atoms at pH 1; **Assay conditions**: Time (days)
**SSO**	8	**Protein descriptor**: No of chain atoms, Minimal projection radius, No of ring bonds, No of aliphatic rings; Formulation characteristics: -; **Plasticizer descriptor**: No of rings, logD at pH 11; **Emulsifier descriptor**: Hyper wiener index, Wiener polarity; **Assay conditions**: -
**SSO**	13	**Protein descriptor**: Length perpendicular to the max area, Minimal projection radius, Length perpendicular to the min area, No of rings; Formulation characteristics: Production method; **Plasticizer descriptor**: No of ring bonds, principal component of polarizability tensor axx, logD at pH 3; **Emulsifier descriptor**: Smallest ring size, Markush library size, No of hydrogen bond donors, No of acceptor atoms at pH 14, No of donor atoms at pH 4; **Assay conditions**: -
**LASSO**	19	**Protein descriptor**: -; Formulation characteristics: Production method; **Plasticizer descriptor**: No of fragment count, logD at pH from 0 to 14, logP; **Emulsifier descriptor**: -; **Assay conditions**: Time (days)
**LASSO**	22	**Protein descriptor**: -; Formulation characteristics: Mean particle size (μm), PLGA to plasticizer ratio, Dissolution pH, Production method; **Plasticizer descriptor**: No of fragment count, logD at pH from 0 to 14, logP; **Emulsifier descriptor**: -; **Assay conditions**: Time (days)

**Table 3:** NRMSE for input vectors selected by bio-inspired algorithms.

FS method	No. Inputs	Cubist	Mon-mlp	MARS	CART	RF	fugeR
**ALO**	8	22.45	24.55	25.90	27.64	21.95	-
	12	25.95	25.15	25.30	26.13	22.19	-
	20	18.73	20.20	21.45	21.30	16.33	20.15
**BALO**	9(1)	21.20	20.63	21.28	24.17	18.81	-
	9(2)	18.26	17.31	20.86	20.99	15.97	18.09
	9(3)	22.60	21.88	22.15	23.80	19.79	-
	11	19.40	19.35	21.37	24.25	18.70	-
	12	17.26	18.17	21.41	22.33	16.56	18.73
**GWO**	15	19.30	18.88	20.64	20.10	16.73	19.10
	18	20.65	18.58	21.34	22.21	17.63	-
	24	20.30	22.30	21.40	22.88	17.90	-
	25	20.04	19.29	19.10	20.50	15.86	19.10
	26	17.32	22.22	21.74	19.97	16.22	-
**SSO**	8	30.49	31.12	30.97	32.60	28.89	-
	13	27.09	25.82	26.45	25.60	24.86	-
**LASSO**	19	22.78	17.90	21.74	21.70	17.15	-
	22	22.78	18.48	21.74	21.59	17.20	-

RF model developed on the nine input vector, 9(2) (purple circle in Fig 2), selected by BALO algorithm, yielded one of the lowest NRMSE. Deep learning neural networks applied to the same vector resulted in error of 16.87%. Selected inputs are shown in Table 4. Comparable results were obtained, 15.97% versus 15.4%, to those by Szlȩk et al. [10], but the vector of inputs was smaller, nine versus eleven. It is a rule of a thumb that increasing the number of independent variables improves the model fitness; however, the model might be unnecessarily complex. In this case, we have reduced the complexity by reducing the number of inputs by two (over 18% reduction of the number of inputs) at the same time NRMSE raised only of 0.6%. It can be noted from Table 4 that the methods such as MARS and CART failed, giving the lowest NRMSEs of 19.10 and 19.97 respectively.

**Table 4:** Results for BALO 9(2), trained and tested on 10cv data sets.

Algorithm	NRMSE	R2
**Cubist**	18.26	0.611
**Monmlp**	17.31	0.652
**Deep learning neural nets**	16.87	0.655
**fugeR**	18.09	0.612
**RF**	15.97	0.692
**CART**	19.97	0.571
**MARS**	19.10	0.591

Both BALO and the method described by Szlȩk et al. [10] lead to the selection of ‘Time (days)’ and ‘PVA inner phase concentration (%)’ as crucial parameters governing the dissolution process. Moreover, as it is depicted in Fig 2, variables ‘PVA inner phase concentration (%)’ (circle labeled as 89), and ‘Time (days)’ (labeled as 300) were also selected by most of the algorithms. Not surprisingly the time was the most common variable for all data sets. The time variable determines dissolution profile both physically and numerically—the latter is due to the structure of data set, where each dissolution point is represented by separate data record. Despite high occurrence of the variables belonging to formulation characteristics, e.g. ‘Encapsulation ratio’ (circle labeled as 93) or ‘Production method’ (labeled as 101), it is worth pointing out that also protein molecular descriptors such as, ‘Minimal projection radius’ (labeled as 35) and ‘No of aliphatic bonds’ (labeled as 7) were present. This may suggest an influence of protein size on its dissolution from PLGA microspheres.

Moreover, BALO algorithm (BALO_9(2)) separated four variables describing peptide drugs and three for excipients. In contrary, Szlȩk et al. [10], have pointed to three inputs representing proteins, but none of them described excipients (Table 5). Depending on the algorithm used, these chemical descriptors are either included or not in the features vector; thus their impact on dissolution is not well established. Further enhancement of the database would be required to elucidate the above relationships.

**Table 5:** Comparison of selected features by BALO and obtained by Szlȩk et al. [10].

BALO 9in(2)	Szlȩk et al. [10] 11in
No of hydrogen atoms–protein descriptor.	Szeged index–protein descriptor.
No of heteroaromatic rings–protein descriptor.	pI–protein descriptor.
No of rings–protein descriptor.	Quaternary structure of macromolecule: 1- monomer, 2–dimer–protein descriptor.
No of heteroaliphatic rings–protein descriptor.	Lactide to Glycolide in polymer ratio—formulation characteristics.
PVA inner phase concentration (%)—formulation characteristics.	PVA inner phase concentration (%)—formulation characteristics.
Heteroaliphatic ring count–plasticizer descriptor.	PVA outer phase concentration (%)—formulation characteristics.
LogD at pH 9–plasticizer descriptor.	Encapsulation rate (%)—formulation characteristics.
No of donor atoms–emulsifier descriptor.	Mean particle size (μm)—formulation characteristics.
Time (days)–assay conditions.	Dissolution pH–assay conditions. Production method: 1–w/o/w, 2–s/o/w, 3–s/o/o,4–spray-dried—formulation characteristics. Time (days)–assay conditions.

**Fig 3:**
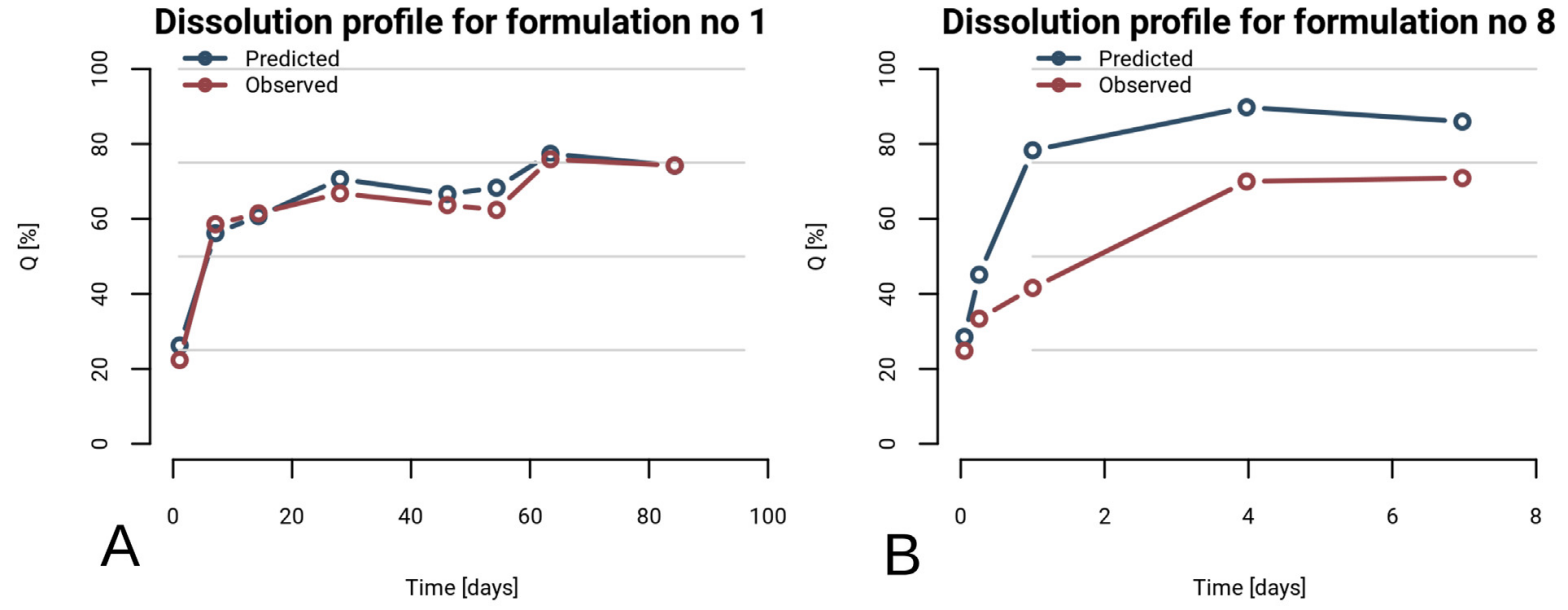
Examples of predicted vs. observed dissolution profiles. RF model was used to predict profile.

Overall RF model’s performance is a result of an average error of separate formulations. As it is depicted in Fig 3, the error may vary and it depends on formulation characteristics. An example of good prediction, which falls below 5%, is presented in Fig 3-A. In contrary, failed prediction is showed in Fig 3-B (NRMSE of 20.6%). Therefore, errors of 10-cv predictions were closely investigated. The NRMSE of 24 formulations fell below 10%, 19 formulations had an error between 10 and 15%, and the rest 25 formulations showed error higher than 15%. It indicates good performance of the model for more than two-thirds tested formulations. When constituents of microspheres were investigated it was observed that, out of 14 proteins, only for four of them predictions failed (chymotrypsin, human serum albumin, insulin, L-asparaginase). Therefore, it is strongly recommended not to use the final model to predict dissolution profiles for PLGA microspheres containing those proteins. The high errors for those formulations could be the result of the small number of formulations consisting those proteins. On the other hand obtained errors for bovine serum albumin, recombinant human erythropoietin, recombinant human epidermal growth factor, lysozyme, recombinant human growth hormone, hen ovalbumin, beta-amyloid, recombinant human erythropoietin coupled with human serum albumin, bovine insulin, alpha-1 antitrypsin were below 15%, which points that the most of the dissolution profiles were well predicted.

Nevertheless the results obtained show that random forest is useful to predict dissolution behavior from PLGA microspheres. Moreover, the results obtained come from a model trained on the use of enhanced 10-cv technique, which may indicate that the model will be useful even if unknown formulations are meant to be predicted.

## 4 Conclusions

In this paper, we address the PLGA feature selection problem by using bio-inspired optimization algorithms such as antlion optimization (ALO), binary version of antlion optimization (BALO), grey wolf optimization (GWO), and social spider optimization (SSO), and also LASSO algorithm to select optimal feature subset for predicting the dissolution profile of PLGA. Feature selection is considered as a biobjective optimization problem that minimizing the prediction error of the data analysis model while minimizing the number of features used. A set of input features were employed to find minimum generalization error across different predictive models and their settings/architectures. We evaluated our proposed solution using different predictive modeling algorithms such as cubist, random forests, artificial neural networks (monotonic MLP, deep learning MLP), MARS, CART, and hybrid systems of fuzzy logic and evolutionary computations (fugeR). The experimental results are compared with Szlȩk results. We obtained a root mean square error 15.97% versus 15.4%, but the selected input features was smaller, nine versus eleven.
